# Supplementary material for: XYLT1 Deficiency of Human Mesenchymal Stem Cells: Impact on Osteogenic, Chondrogenic, and Adipogenic Differentiation
Source: Int J Mol Sci. 2025 Jul 30;26(15):7363. doi: 10.3390/ijms26157363 (PMC12347940; doi:10.3390/ijms26157363)
Supplement: Supplementary file 1 [file ijms-26-07363-s001.zip › ijms-3741875-supplementary.pdf]

Supplementary material

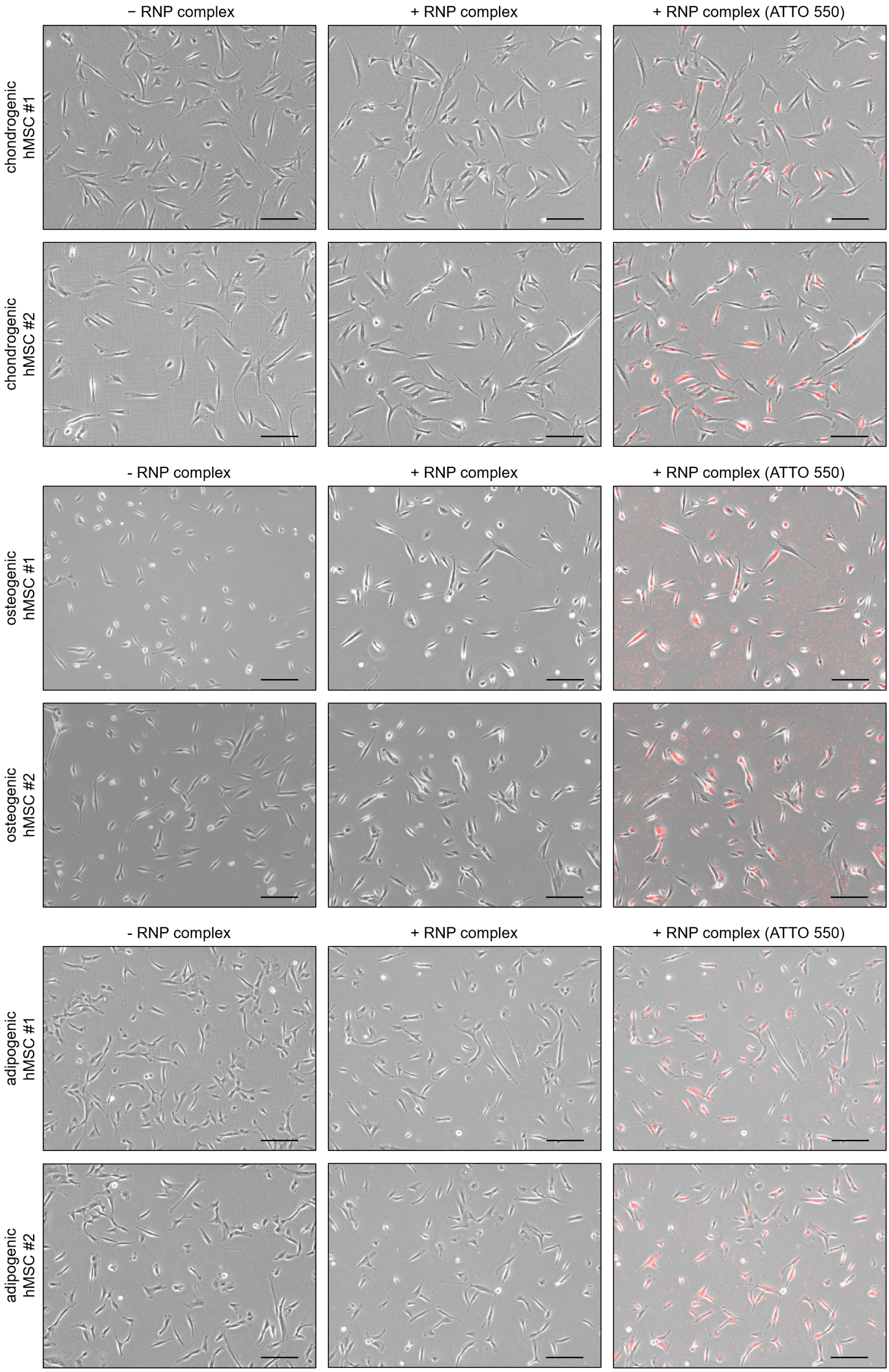

Fig. S1: **Transfection efficiency of the RNP-based CRISPR-Cas9 system in KD hMSC cultures determined by ATTO 550-labeled tracrRNA**. Representative images of hMSC cultures 24 h post-transfection of the CRISPR-Cas9 RNP system prior to tri-lineage differentiation. Scale bar represents 100 µm.

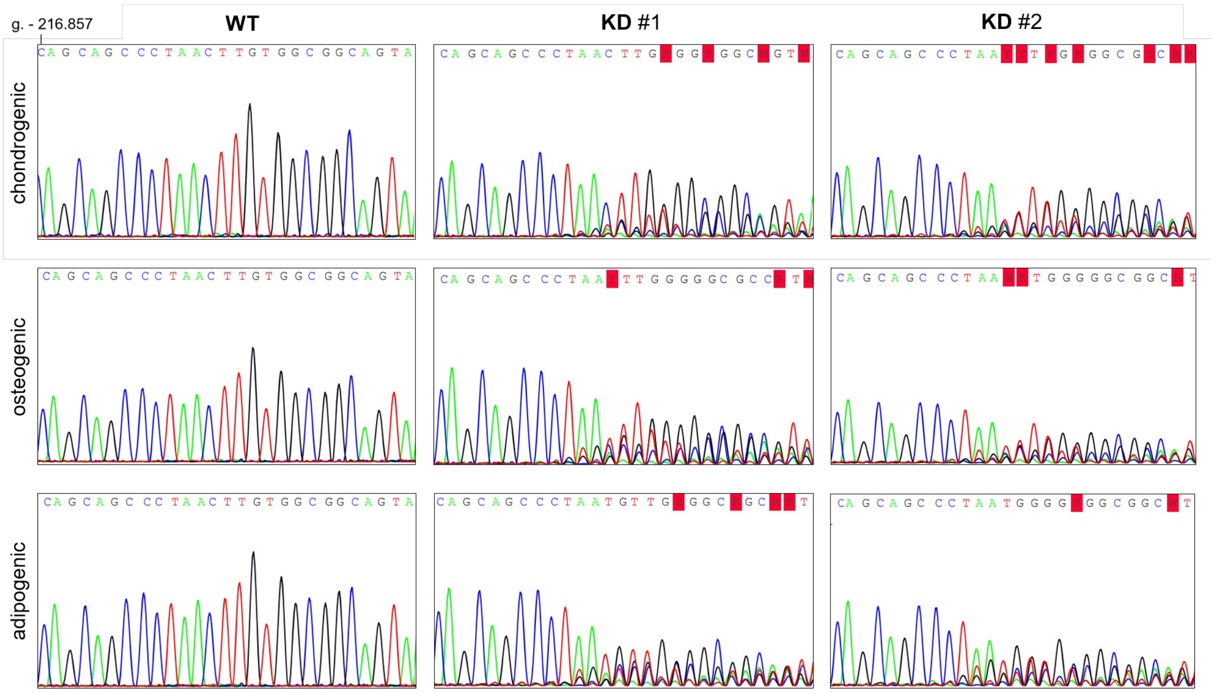

Fig. S2: Sequencing results of all CRISPR/Cas9 experiments targeting exon 3 of the *XYLT1* gene (Gene ID: 64131)**.** The primary hMSC cultures (passage 3) were mock transfected (WT) or transfected with the complete RNP complex (KD). DNA was isolated from hMSC cultures (#1, #2) 72 h post-transfection, before further assessing basal expression and chondrogenic, osteogenic, or adipogenic differentiation.

g. - 216.859

	PAM / gRNA		
**Reference NC_000016.10**	atcagcag[ccc][taacttgtggcggcagtaag]tctc		959 AS
Clone m2	atcagcagccctaa--tgtggcggcagtaagtctc	2 bp deletion	293 AS
Clone m7	atcagcagccctaactt gtggcggcagtaagtctc (↑ t)	1 bp insertion	294 AS
Clone m8	atcagcagccct----tgtggcggcagtaagtctc	4 bp deletion	290 AS
Clone m9	atcagca ------- cttgtggcggcagtaagtctc	7 bp deletion	289 AS
Clone m19	atcagcagccctaa---gtggcggcagtaagtctc	3 bp deletion	253 AS
Clone m20	atcagcagccctaactt gtggcggcagtaagtctc (↑ a)	1 bp insertion	294 AS
Clone m21	atcagcagccct----tgtggcggcagtaagtctc	4 bp deletion	290 AS
Clone m22	atccgcagccctaactt gtggcggcagtaagtctc (↑ a)	1 bp insertion<br>1 bp exchange	294 AS
Clone m23	atcagcagccctaactt gtggcggcagtaagtctc (↑ t)	1 bp insertion	294 AS
Clone m26	atccgcagccctaactt gtggcggcagtaagtctc (↑ a)	1 bp insertion<br>1 bp exchange	294 AS
Clone m27	atcagca ------- cgagaagcggc----------<br>--------------------------------------	55 bp deletion<br>4 bp exchange	273 AS
Clone m28	atcagcagccctaa--tgtggcggcagtaagtctc	2 bp deletion	293 AS
Clone f2	atcagcagccctaa--tgtggcggcagtaagtctc	2 bp deletion	293 AS
Clone f4	atcagcagccctaactt gtggcggcagtaagtctc (↑ a)	1 bp insertion	294 AS
Clone f10	atcagcagcc------tgtggcggcagtaagtctc	6 bp Deletion	289 AS
Clone f12	atcagcagccctaa--tgtggcggcagtaagtctc	2 bp Deletion	293 AS
Clone f21	atcagcagcct taacttgtggcggcagtaagtctc	1 bp Austausch	253 AS
Clone f22, f23 & f27	atcagcagccctaacttgtggcggcagtaagtctc (↑ a)	1 bp Insertion	294 AS

Fig. S3: Validation of CRISPR-Cas9 editing events in hMSC populations by TA cloning. The primary hMSC cultures (passage 3) were mock transfected (WT) or transfected with the complete RNP complex (KD). DNA was isolated from hMSC cultures (#1, #2) 72 h post-transfection. TA cloning was employed to confirm and characterize the editing events in various heterogenous hMSC populations following CRISPR-Cas9 intervention before further assessing basal expression and chondrogenic, osteogenic, or adipogenic differentiation. The depicted sequence segments start at position -216,855 (GenBank accession ID NG_015843.2). The *XYLT1* gRNA sequence (blue) for complementary binding in exon 3 and the PAM sequence (red) required for Cas9 binding are highlighted. Next to each sequence segment, the mutation and the resulting truncation of the XT-I amino acid sequence are indicated.

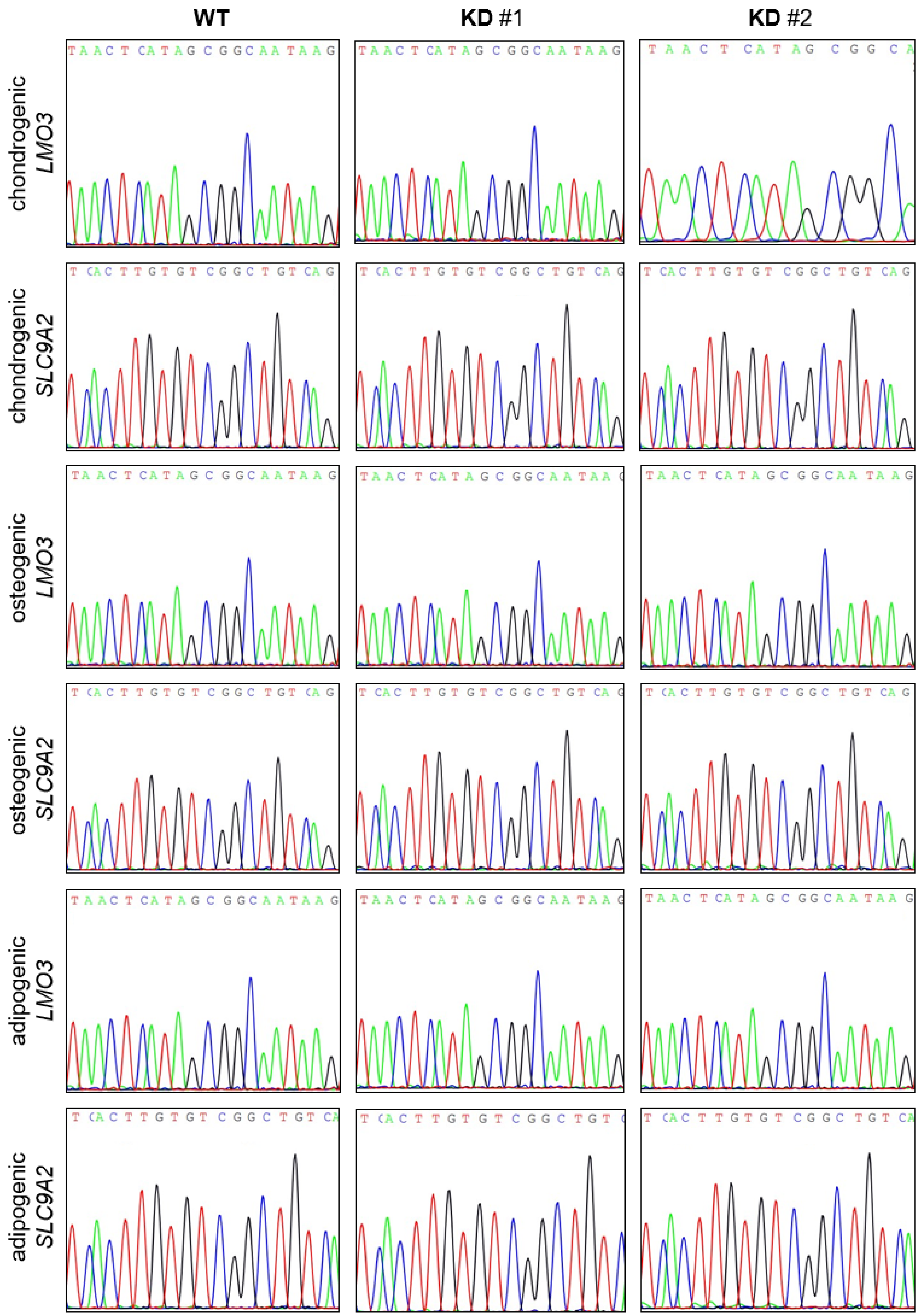

Fig. S4: Analysis of off-target sequences in *LMO3* and *SLC9A2* genes for the utilized *XYLT1* gRNA. Sequencing results of off-target sites within the *LMO3* and *SLC9A2* genes. The displayed sequence fragments start at position g. -59,552 (*LMO3*; GenBank accession ID: NC_000012.12) and position g. -90,054 (*SLC9A2*; GenBank accession ID: NG_050930.1). The primary hMSC (passage 3) were mock transfected (WT) or transfected with the complete RNP complex (KD). DNA was isolated from hMSC cultures 72 h post-transfection, before further assessing basal expression and chondrogenic, osteogenic, or adipogenic differentiation.

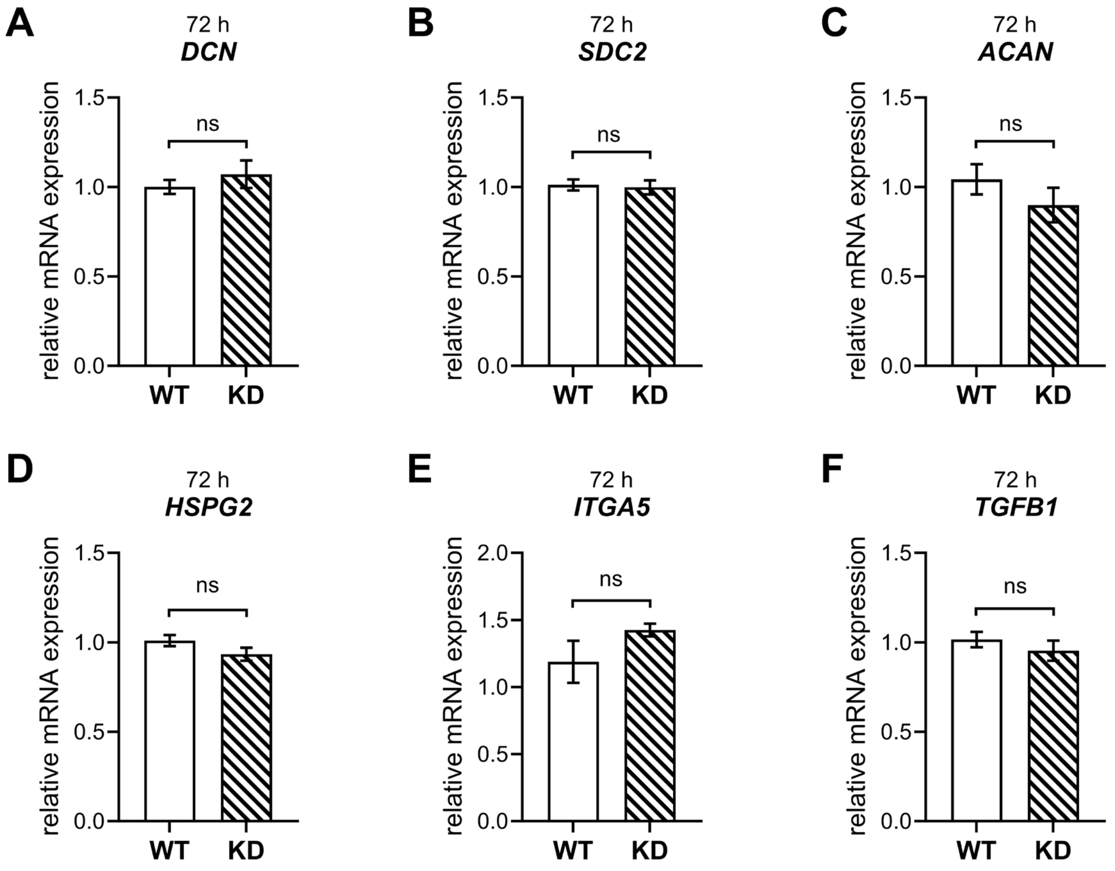

Fig. S5: No significant differences in ECM-related gene expression levels between WT and KD hMSCs. Primary hMSCs were transfected either without (WT) or with (KD) the RNP complex and cultured until reaching 90 % confluence. The cells were subcultured at a density of 50 cells/mm^2 and grown for 72 h prior to cell lysis and sample preparation for gene expression analysis. The relative mRNA expression level of (**A**) *DCN*, (**B**) *SDC2,* (**C**) *ACAN*, (**D**) *HSPG2*, (**E**) *ITGA5* and (**F**) *TGFB1* was analyzed using qRT-PCR, with results displayed as means ± SEM from n = 2 primary cell cultures, n = 3 biological replicates per primary cell culture and n = 3 technical replicates per biological replicate . Statistical analysis indicates no significant differences between the *XYLT1*-deficient and control groups, as assessed by the Mann-Whitney U test (ns = not significant).

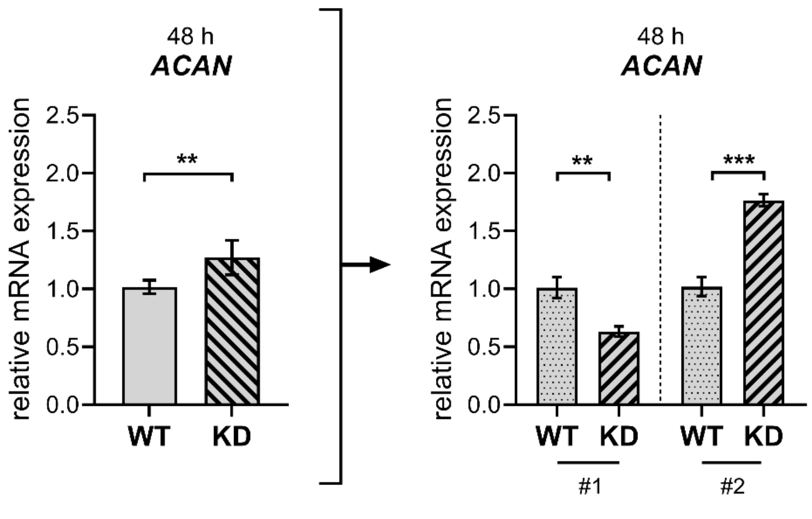

Fig. S6: ***XYLT1* deficiency leads to aberrant *ACAN* expression in individual chondrogenic differentiation experiments**. The hMSCs were cultured to 90 % confluence following transfection without (WT) or with (KD) the

RNP complex. Chondrogenic differentiation of hMSCs was performed using a high-density pellet culture with 250,000 cells maintained either in chondrogenic differentiation medium. Gene expression analysis upon early chondrogenesis was performed at 48 h by determining the mRNA levels of *ACAN* via qRT-PCR. Data are means ± SEM from n = 2 primary cell cultures, n = 3 biological replicates per primary cell culture and n = 3 technical replicates per biological replicate, normalized to the WT sample. Left side shows data from both experimental procedures (n=2 primary cell cultures), while right side shows the data from individual experiments (#1, #2). Mann-Whitney test significance levels: $p < 0.01$ (**), $p < 0.001$ (***).

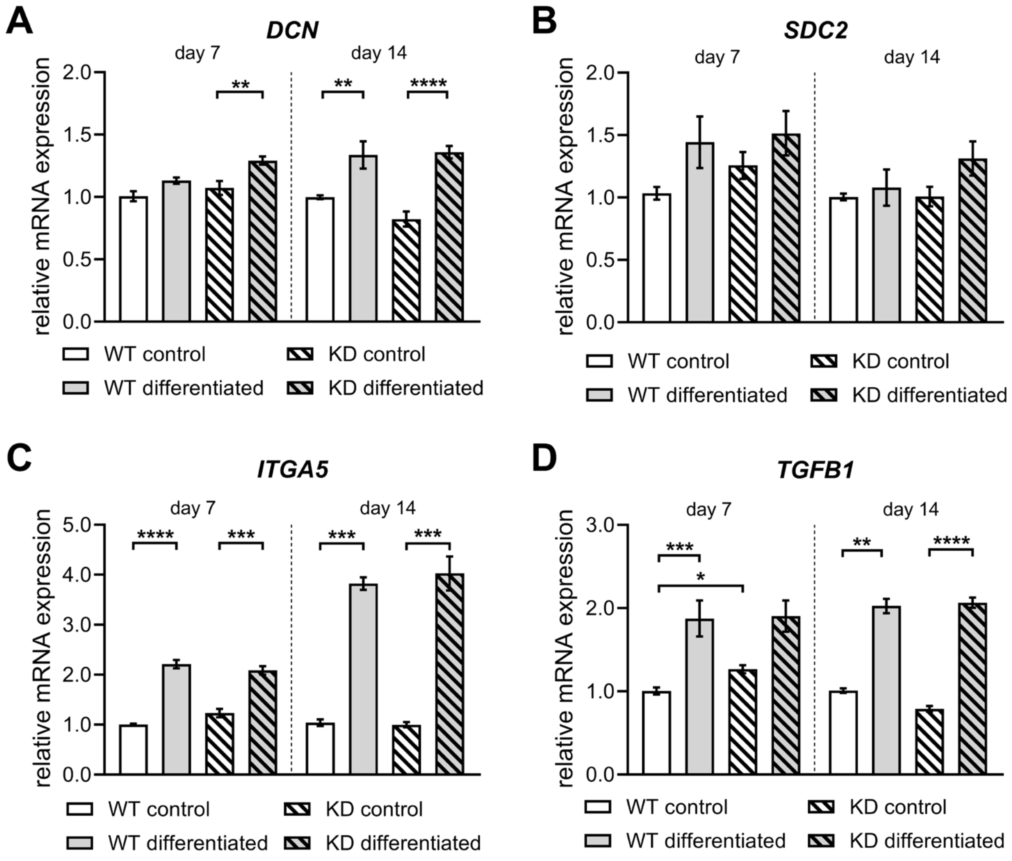

Fig. S7: **Analysis of *DCN*, *SDC2, ITGA5* and *TGFB1* expression in osteogenic differentiated WT and KD hMSC cultures**. Primary hMSCs were transfected either without (WT) or with (KD) the RNP complex and cultured until reaching 90 % confluence. The cells were subcultured at a density of 100 cells/mm^2, grown until 100 % confluence, and then cultivated for 7 or 14 days in either hMSC standard medium (control) or osteogenic differentiation medium (differentiated). The mRNA expression levels of (**A**) *DCN*, (**B**) *SDC2*, and (**C**) *ITGA5* and (**D**) *TGFB1* was determined at both day 7 and day 14 of osteogenic differentiation. Data are presented as means ± SEM from n = 2 primary cell cultures, n = 3 biological replicates per primary cell culture and n = 3 technical replicates per biological replicate, with normalization to the respective WT control. Kruskal-Wallis significance levels: $p < 0.05$ (*), $p < 0.01$ (**), $p < 0.001$ (***), and $p < 0.0001$ (****).

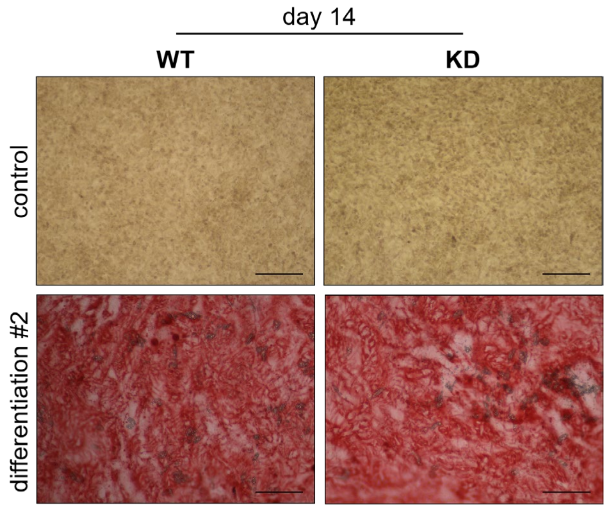

Fig. S8: **Analysis of calcium depositions in osteogenic differentiated WT and KD hMSC cultures.** hMSCs were cultured post-transfection without (WT) or with (KD) RNP complex until reaching 90 % confluence. Cells were subcultured at a density of 100 cells/mm² and, upon reaching 100 % confluence, cultured for 14 days in either hMSC standard medium (control) or osteogenic differentiation medium (differentiation). Shown are representative images of WT and KD hMSCs cultures on day 14. Scale bar: 100 µm.

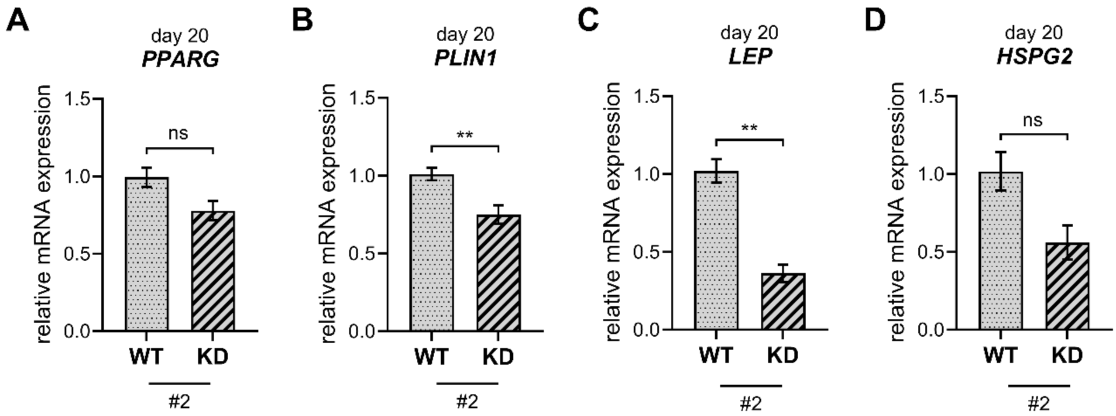

Fig. S9: **Relative quantification of mRNA expression levels of adipocyte markers in adipogenic differentiated WT and KD hMSC cultures.** The hMSCs were cultured post-transfection with (KD) or without (WT) the RNP complex until reaching 90 % confluency. Cells were seeded at a density of 210 cells/mm² and cultured in standard hMSC medium until 100 % confluency. Adipogenesis was induced over a 20-day period through three cycles of three days in induction medium followed by one day in maintenance medium. Subsequently, cells were cultured in maintenance medium for an additional seven days. The mRNA expression level of (**A**) *PPARG*, (**B**) *PLIN1*, (**C**) *LEP*, and (**D**) *HSPG2* was determined via qRT-PCR. Data are means ± SEM from n = 1 primary cell culture, n = 2 biological replicates per primary cell culture and n = 3 technical replicates per biological replicate, normalized to the WT control. Mann-Whitney test significance levels: not significant (ns), $p < 0.01$ (**).
